# Safety of 200iU of intravesical Botox in children of all ages with functional urinary incontinence

**DOI:** 10.1007/s00383-025-06106-7

**Published:** 2025-07-10

**Authors:** Eliza Szwarcberg, Chris Kimber, Kiarash Taghavi

**Affiliations:** 1 Department of Paediatric Urology, Monash Children’s Hospital, 246 Clayton Rd, Clayton, Melbourne, VIC 3168 Australia; 2 Department of Paediatrics, School of Clinical Sciences, Faculty of Medicine, Nursing and Health Sciences, Monash University, Melbourne, Australia

**Keywords:** Urinary incontinence, Botulinum toxins, Type A, Paediatrics

## Abstract

**Purpose:**

Intravesical Botox injection can be effective in children with functional urinary incontinence refractory to conservative measures. A wide range of potential doses have been reported with concerns regarding the safety of higher doses. This study aims to determine the safety and side effect profile of 200iU of Botox in children of all ages with functional urinary incontinence.

**Methods:**

A retrospective review of 143 children who received 200iU of botulinum toxin A utilising intradetrusor injections was performed. All children had urinary incontinence with no defined neurological abnormality. Post-operative complications were analysed, including UTI, adverse drug reactions, pain, haematuria, urinary retention, transitory increased incontinence and systemic adverse effects.

**Results:**

A total of 228 injection procedures (range: 1–6 procedures) in 143 children (70 girls, 73 boys) were included. Median age at first treatment was 9 years (range: 4–17 years). The mean number of treatment cycles required per patient was 1.59 (range: 1–6). There were no intra-operative complications. Post-operative complications included UTI, post-operative pain and haematuria. No complication required re-admission and no cases of urinary retention requiring catheterisation occurred.

**Conclusion:**

This study represents the largest population to date of children with functional urinary incontinence treated with intravesical Botox, confirming this as a well-tolerated, safe intervention in all ages with complications as would be expected for cystoscopy alone.

**Supplementary Information:**

The online version contains supplementary material available at 10.1007/s00383-025-06106-7.

## Introduction

*Onabotulinum toxin A* (Botox, Allergan, Irvine, CA, USA) is commonly used as an intravesical injection for children with functional urinary incontinence. Behavioural therapy and antimuscarinic agents are the mainstay of treatment for daytime lower urinary tract conditions. However, response to these methods varies and many children experience significant side effects. [1] Previous studies have demonstrated that intravesical Botox injection is a well-tolerated, safe and effective intervention in these refractory cases. [2–9] In children with lower urinary tract dysfunction resistant to urotherapy and oral agents, intravesical Botox has shown 50–70% success following one treatment in a small case series. [9] Additionally, optimal dosing of intravesical Botox injection in the context of functional urinary incontinence in children remains debated.

Previous studies have used a wide range of doses, including 100iU and 10–12.5iU/kg with maximum dose of up to 300iU. [2–9] In children with neurogenic bladder, it has been demonstrated that 200iU has greater efficacy compared to 50iU or 100iU while maintaining a similar safety profile. [10] Urinary retention is the most concerning side effect, and if persistent may necessitate intermittent catheterisation to facilitate bladder emptying. Other post-operative complications observed in some studies include urinary tract infection (UTI) and transient frank haematuria. There is concern for potential systemic effects such as musculoskeletal weakness and breathing difficulties, though no cases have previously been reported. It is important to confirm a safe dose for clinical use in children with functional urinary incontinence. The present study incorporated the largest series of children with functional urinary incontinence treated with intravesical Botox to date, aiming to determine the safety and side effect profile of 200iU intravesical Botox injections in children with non-neurogenic bladder dysfunction of all ages.

## Materials and methods

The medical records of all children who underwent intravesical Botox injection by one surgeon between January 2018 and December 2023 were retrospectively audited. All children included in this study had urinary incontinence with no known underlying neurological abnormality (on a clinical and/or radiological basis). Invasive cystometrography was not performed pre-operatively due to the invasive nature of this investigation performed awake. Uroflowmetry and renal tract ultrasound (with pre- and post-void bladder volumes) were performed on a case-by-case basis, but treatment decisions were made predominantly based on clinical grounds and bladder diary reports. No child in this series receiving intravesical Botox alone had significant post-void residual volumes pre-operatively.

All children received a total of 200iU of *Onabotulinum toxin A* (Botox, Allergan, Irvine, CA, USA) sourced through the hospital pharmacy. Rigid cystoscopy was performed with a Storz Paediatric Cystoscope (9.5Fr). No urethral catheterisation or dilatation was required in any patients. A Williams Cystoscopic Injection Needle (Cook Medical) was utilised for intramuscular injection, which has a 23G needle tip, and 8 mm tip.

The product was diluted into 20 mL of normal saline with a sterile technique, and injected in twenty 1 mL aliquots. The technique utilised intramuscular (detrusor) injections (up to a 6 mm depth) commencing in the midline trigonal region expanding over the base and dome of the bladder (avoiding the ureteric orifices and the ureteric intravesical course). The intention was to deposit each aliquot predominantly within the detrusor muscle itself rather than submucosally. A video demonstrating the described technique is available to be viewed as supplementary content to this article. All children received a 25 mg/kg dose of intravenous cephazolin at induction. The treatment was performed under general anaesthesia in all cases.

**Fig. 1:**
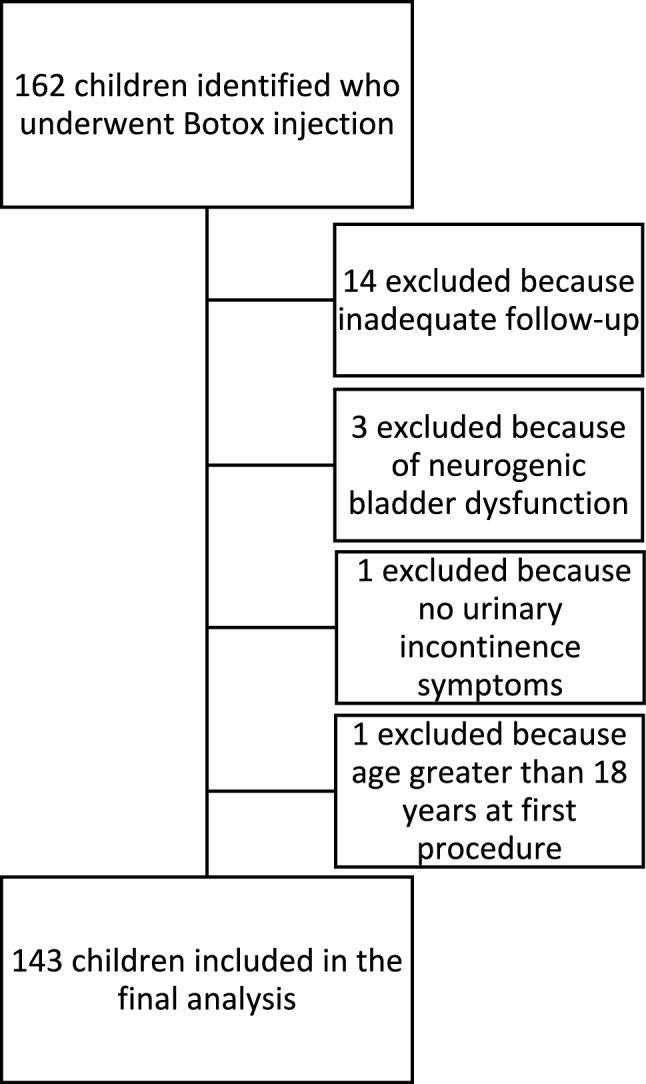
Flow diagram indicating inclusion and exclusion of patients to give final cohort

Overall, 162 children underwent intravesical Botox injection over the study period. Of these, 143 children (70 females and 73 males) were included in this analysis (Fig. 1). All children had previously trialled alternative treatment options including pharmacotherapy and behavioural therapies before receiving Botox injection.

If children were on multi-modal therapy (e.g. oxybutynin, mirabegron) pre-operatively, this was ceased in the post-operative period and all children were routinely reviewed at 4 weeks post-operatively. At this point clinical response was assessed, with selective use of a bladder diary and uroflowmetry. The appointments were conducted by the treating surgeon either in person or via telehealth. Subsequent ongoing follow-up and investigations were arranged on a case-by-case basis depending on clinical course. Post-operative complications were analysed, including rates of UTIs, adverse drug reactions, pain, haematuria, urinary retention, transitory increased incontinence and systemic adverse effects such as musculoskeletal weakness and breathing difficulties following Botox injection. Symptomatic UTIs were attributed to Botox injection if symptom onset was within the two-week post-operative period. Further studies are planned to present the efficacy of treatment in each individual diagnostic group.

## Results

A total of 228 injection procedures were performed in 143 children for functional urinary incontinence between January 2018 and December 2023. Median age at first treatment was 9 years (range 4–17 years). The ICCS definition requires a minimum age of 5 years for the diagnosis of functional bladder dysfunction. However, a single child aged 4 years and 11 months old was included in the cohort to appropriately reflect the practice experience [11]. The indication for Botox injection as determined by presenting symptoms and as per ICCS definitions is presented in Table 1. Clinical symptoms of bladder overactivity included urinary urgency and/or bladder spasms. Multiple pre-operative UTIs had occurred in 15 children, of whom five had co-existent vesicoureteric reflux. Co-existing neuropsychiatric disorders were present in 8% of children (12/143), including Attention-deficit/hyperactivity disorder (ADHD) in 7, Autism Spectrum Disorder (ASD) in 1, Intellectual Disability in 1, and ASD and ADHD in 3 children.

**Table 1:** Breakdown of diagnosis for functional bladder dysfunction as per ICCS definitions

Symptoms	% (*n*)	Median Age (range)
Daytime incontinence	66% (94)	8 (4–16)
Non-monosymptomatic nocturnal enuresis	55% (79)	8 (4–16)
Clinical symptoms of bladder overactivity	29% (41)	8 (4–18)
Severe urinary frequency	21% (30)	9 (4–18)
Monosymptomatic nocturnal enuresis	19% (27)	12 (7–14)
Giggle incontinence	2% (3)	9 (7–11)

**Fig. 2:**
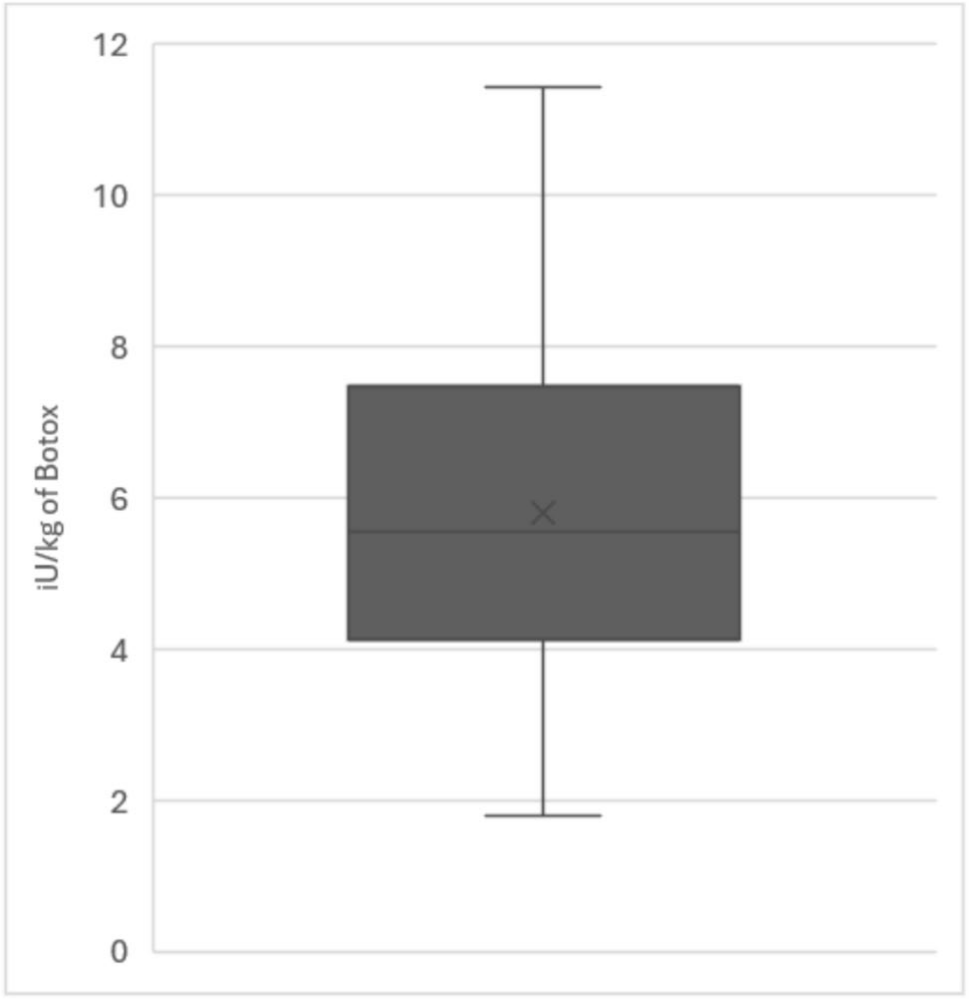
Distribution of dosage per kilogramm of Botox delivered to the study population

Data regarding the weights of study participants at the time of procedure was available in 71% of cases. The median dosage per kilogram of Botox delivered to these children was 5.6 units per kilogram (range: 1.8–11.4, distribution displayed in Fig. 2). The mean number of treatment cycles required per patient was 1.59 (range: 1–6, detailed data in Table 2). The mean interval between treatment cycles was 14 months (range: 4–31 months).

**Table 2:** Number of total treatment cycles required in children treated with 200iU of Botox with functional bladder disorders

Number of injection procedures	% (*n*)
1	58% (83)
2	31% (44)
3	7% (10)
4	3% (4)
5	1% (1)
6	1% (1)

There were no intra-operative complications recorded. Post-operative complications reported included UTI, post-operative pain and haematuria (detailed data in Table 3).

**Table 3:** Breakdown of adverse events following the injection procedure

Adverse event	% (*n*)
Urinary tract infection	1% (3)
Pain	1% (3)
Haematuria	1% (2)
Adverse drug reaction	0%
Urinary retention	0%
Worsening transitory incontinence	0%
Systemic effects	0%

Three children (all female) experienced a symptomatic UTI following the procedure (none requiring admission). None of these children had a previous history of recurrent UTIs. One child suffered from a single UTI after treatment without further complications. Recurrent UTIs occurred in a 6-year-old girl, with the first UTI one week post-injection. One girl had a culture-positive UTI during the first two weeks post-injection, which was further complicated by suprapubic pain and macroscopic haematuria. She had ongoing suprapubic pain for several months which had resolved on review at 12 months.

Three children, all female, had post-operative pain managed with oral analgesia and not requiring re-admission. One 13-year-old girl had associated intermittent difficulty voiding, but never had a significant post-void residual volume (> 30 mL) and did not require catheterisation. Her symptoms gradually resolved by three months post-op. One girl had two days of macroscopic haematuria which self-resolved.

There was no transitory increased incontinence following the procedure, although this has previously been reported as a potential complication of intravesical Botox injection. No patients experienced post-operative urinary retention requiring catheterisation. No patients required re-admission.

## Discussion

Intravesical Botox injection has been demonstrated to be an effective intervention in children with functional urinary incontinence refractory to more conservative measures. The present study investigated the safety of 200iU intravesical Botox injection in children of all ages. This represents the largest experience of children with functional urinary incontinence treated with intravesical Botox injection to date, confirming the procedure as a well-tolerated and safe management option and establishing a near zero risk of urinary retention requiring intermittent catheterisation. All complications occurred following the child’s first therapy, suggesting that repeat injections are as safe as the initial injection.

Botulinum toxin is a neurotoxin produced by the *Clostridium* genus. Intravesical administration has possible mechanisms of action through both efferent and afferent pathways. The former acts by decreasing muscle contractility and blocking the release of acetylcholine. The latter acts via an inhibitory effect on neurotransmitters and receptors that mediate sensory neurotransmission.

While the anticholinergic properties of onabotulinum toxin A may have been the initial rationale for its use in detrusor overactivity, onabotulinum toxin A affects the expression and release of other substances in the bladder. When the bladder is exposed to stressors, onabotulinum toxin A improves compliance by downregulating the release of adenosine triphosphate and upregulating the release of nitrous oxide [12]. Additionally, it affects the sensory pathways of the bladder by desensitising unmyelinated C-fibres in the urothelium [13]. Intradetrusor injection is thought to act via inhibition of presynaptic release of acetylcholine, resulting in chemo-denervation and paralysis [14].

Findings in the study were consistent with the safety profile of intravesical Botox injection in children with functional urinary incontinence demonstrated previously in smaller studies. Some previous studies have reported cases of temporary urinary retention needing intermittent catheterisation to facilitate bladder emptying [3–5]. A summary of these cases are presented in Table 4. Importantly, there were no cases of urinary retention in the current study. Hoebeke et al. reported one girl who received 100iU Botox and experienced 10 days of urinary retention requiring intermittent catheterisation, after which voiding normalised [5]. In another study, one child went into urinary retention post-operatively ultimately requiring a suprapubic catheter for 2 months. This patient received intravesical Dysport injection with a dose exceeding 20iU/kg body weight [3]. Greer et al. reported 15 children with non-neurogenic bladder dysfunction who received intravesical Botox injections of 10iU/kg up to a maximum of 300iU. Of these, 2 children (5%) presented to hospital with urinary retention [4]. The higher rate of urinary retention in this study may be attributed to the increased dose of botulinum toxin A (10iU/kg, max 300iU), although the exact dose received by those children who experienced urinary retention was not reported. Finally, it may be that the low rate of urinary retention in the current large case series (when compared to published literature) relates to injection technique. Submucosal injection may impact afferent neural pathways more dominantly with a higher consequent risk of urinary retention, although this is speculative [15].

**Table 4:** Summary of previously reported cases of urinary retention in children with functional urinary incontinence following intravesical Botox injection

Study	Sample size	Dose used	Urinary retention
Hoebeke et al. [5]	*n* = 21	100iU Botox	1 girl requiring 10 days CIC
Blackburn et al. [3]	*n* = 27	> 20iU/kg Dysport	1 child requiring 2 months suprapubic catheter
Greer et al. [4]	*n* = 15	10iU/kg Botox, max 300iU	2 children (4.88%) presented to emergency department in retention

*CIC* clean intermittent catheterisation

No children in this study experienced anaphylactoid, locoregional or systemic effects from Botox administration. Importantly, the lethal dose of botulinum toxin A is 39 to 49 iU/kg injected intravascularly [16]. Therapeutic botulinum toxin A is not injected intravascularly and is used at much lower doses, with most urological applications not exceeding 300iU. Other potential systemic side effects included mild generalised weakness and temporary autonomic side effects. To date, this has not been reported in paediatric patients receiving intravesical Botox injection for functional urinary incontinence. Minimising the injected volume may be important as the risk of systemic absorption and generalised weakness appears to be more dependent on the volume than the quantity of botulinum toxin A [17].

Other post-operative complications observed included UTI, temporary haematuria and post-operative pain. No child with a post-operative UTI had a previous history of recurrent UTI. One girl had reported a single pre-operative UTI. Some previous studies have investigated whether incomplete bladder emptying following the procedure contributes to infection risk. Hoebeke et al. reported normal uroflowmetry and complete bladder emptying in the 2 girls who had a post-operative UTI [5]. Similarly, Bayrak et al. calculated post-void residual urine volume as < 20 mL in the 6 children who had a UTI [2]. Therefore, post-operative infections may not be attributable to the direct effect of botulinum toxin on bladder emptying. Current literature suggests the incidence of post-cystoscopy UTIs in adults ranges from 1 to 9% [18–23]. In children, post-procedural UTIs following voiding cystourethrogram has been reported in up to 10% of patients [24]. This probably explains the role of instrumentation alone as the cause of post-operative UTIs.

There is limited data available to inform the use of peri-operative antibiotics to prevent UTIs following paediatric genitourinary procedures, with studies demonstrating wide variations in practice among paediatric urologists [25, 26]. It is unclear whether prophylactic antibiotics are indicated in this setting, and there is a current multi-centre study planned to illuminate this [27]. It has been shown that previous history of UTI and urological malformations increase the risk of post-operative UTI and some guidelines recommend selective use of antibiotics in these children [28].

There are several limitations of the current study. The retrospective design may underestimate some of the reported complications. After initial follow-up at four weeks post-procedure, children were reviewed in an individualised manner. Furthermore, investigator-gathered complaints and outcomes may also be a source of bias. These factors may have introduced reporting or selection bias; however, those needing hospital admission or emergency department re-attendance are unlikely to be missed. Future studies are needed to investigate whether the use of 200iU Botox has improved efficacy compared to alternative dosing options such as 100iU or 10iU/kg body weight. Efficacy studies are also planned to understand if there are patient factors that will predict treatment response.

## Conclusions

This study demonstrates that 200iU of intravesical Botox is a safe dose in clinical treatment of children with functional urinary incontinence of all ages. This is the largest series of children with functional urinary incontinence treated with intravesical Botox injection to date. There were no cases of urinary retention and a low overall complication rate. There is a need for further studies into the efficacy of 200iU as compared to other doses to aid in determining the optimal dose.

## Supplementary Information

Below is the link to the electronic supplementary material.

Supplementary file1 (MP4 39512 KB)

## Data Availability

The data sets generated during and/or analysed during the current study are available from the corresponding author on reasonable request.
